# Assessment of the self‐healing capacity of cementitious materials through active thin sections

**DOI:** 10.1111/jmi.13082

**Published:** 2021-12-28

**Authors:** Emanuele Rossi, Claudia Romero Rodriguez, Henk Jonkers, Oğuzhan Çopuroğlu

**Affiliations:** ^1^ Faculty of Civil Engineering & Geosciences, Department of Materials & Environment Delft University of Technology Delft The Netherlands

**Keywords:** active thin sections, self‐healing

## Abstract

Since self‐healing of cementitious materials can theoretically improve the service‐life of concrete structures, it has gathered significant attention from both researchers and industry during the last two decades. Many researchers have proposed different methods to assess and quantify the self‐healing capacity (i.e. the ability of cementitious materials to heal cracks) that is generated in concrete autogenously as well as autonomously. Even though many methodologies can be found in the literature, a way to accurately quantify the healing products produced by any self‐healing mechanism has not been yet achieved. In this study, a methodology is proposed to observe and to quantify in‐time formation of healing products based on active thin sections. Thin sections of Portland cement paste have been prepared with no epoxy impregnation to facilitate reactions between the cement matrix and the surrounding environment. Artificial cracks (260 μm wide) were induced at 28 days of age and the crystal growth was continuously monitored up to 28 days of self‐healing. Through image analysis of the micrographs, it was calculated that the autogenous self‐healing capacity of paste (triggered by portlandite carbonation in uncontrolled indoor conditions) was around 55% after 28 days of self‐healing. Healing products were further characterised through Environmental Scanning Electron Microscope analysis. Based on the results obtained in this study, the proposed methodology seems to be promising to compare the self‐healing mechanisms triggered by different healing agents.

## 1 INTRODUCTION

To improve the durability of cement‐based materials, self‐healing has drawn quite some interest from both industry and scientific communities during the last decades. Due to the relatively low tensile strength of concrete, cracks are an unavoidable phenomenon affecting concrete structures during their service life. However, concrete has an intrinsic autogenous ability to heal cracks.^1^ Autogenous healing mainly occurs through continuous hydration of cement particles and through precipitation of calcium carbonate resulting from carbonation of the matrix.^2^ Nevertheless, autogenous healing has a healing capacity limited to cracks 0.1–0.2 mm wide.^3^, ^4^, ^5^ Self‐healing capacity of concrete can be improved thanks to several technologies,^5^ such as incorporating fibres,^6^, ^7^ superabsorbent polymers,^8^, ^9^ bacteria‐based particles^10^, ^11^, ^12^ and encapsulated healing agents.^13^, ^14^, ^15^

To investigate the occurrence of the autogenous self‐healing mechanism and that induced by different agents, many methods have been proposed in the literature. Currently, the self‐healing capacity of engineered cementitious materials is generally assessed by two main methods, which are based on observing crack closure during time and by evaluating the recovery of functional properties of interest (i.e. water tightness).^16^, ^17^ Even though the proposed methodologies help to compare some parameters inherent to the self‐healing mechanism as well as the magnitude of recovery of the functional property, there are still some limitations about the accuracy of the healing products quantification. For instance, by measuring the recovery of certain functional properties of the material, the self‐healing capacity is measured indirectly and no data about the amount of the forming healing products are obtained. On the contrary, through imaging‐based methodologies (i.e. surface crack closure through light microscopy), the reduction of crack width during time can be observed. However, in the latter case, quantification of the produced healing products can be hardly conducted. To overcome these limitations, we propose a methodology to observe micro‐scale self‐healing mechanism of cementitious materials that are artificially cracked, through which the amount of precipitates produced by the triggering self‐healing technology can be quantified and observed during time. This methodology is based on active thin sections. Active thin sections have been used in the past by others to evaluate the formation of portlandite crystals and crack patterns through C‐S‐H,^18^, ^19^ as well as to observe the development of very early hydration process of cementitious composites.^20^ The principle behind active thin sections is that 30–50 μm thick layers of cement paste, glued on an object glass, are monitored during time through optical microscopy analysis under polarised light. The material does not get impregnated with fluorescent epoxy resin, which is normally used to preserve the sample integrity. As a consequence, the reaction products generated due to self‐healing at specific locations are observable during time.

## 2 MATERIALS AND METHODOLOGY

**FIGURE 1:**
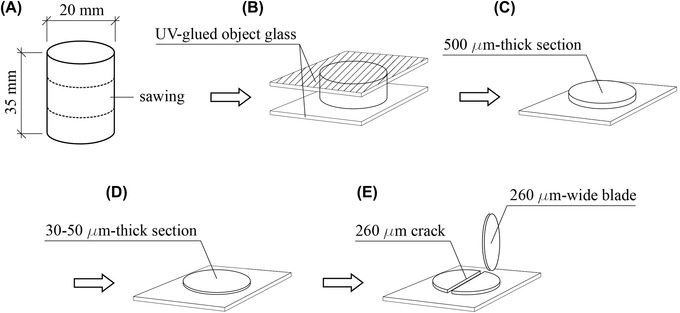
Schematic representation of active thin sections preparation: (A) cement paste cylinders; (B) paste disks with object glasses UV‐glued at both sides; (C) singular 500 μm thick section after sawing; (D) 30–50 μm thick section after grinding with 125 μm and 30 μm diamond plates; (E) thin section with 260 μm wide artificial cracks induced by high‐precision sawing machine

To evaluate the feasibility of analysing self‐healing capacity of cementitious materials through active thin sections, Ordinary Portland cement paste specimens were prepared with a water/cement ratio (*w*/*c*) of 0.50. Paste cylinders of 20 mm in diameter and 35 mm high were cast in plastic moulds, sealed with plastic foil and kept hydrating in a high humidity chamber (RH > 95%) for 28 days. At 28 days of hydration, the specimens were de‐moulded and thin sections were prepared as schematically represented in Figure 1.

The specimens were sawn perpendicularly to their height to obtain about 5 mm thick paste disks. The two faces of the cylindrical disks were glued to a glass object holder with UV activated glue. After glue hardening, the disks were sawn at their middle height with a 2 mm thick sawing blade. As a consequence, two disks of around 1 mm thick could be obtained out of each cylinder. Using a ‘Struers SystemAbele’ grinding machine, the thickness of the disks was firstly manually reduced to around 200 μm with a 125 μm diamond plate and then it was brought to 30–50 μm through a 35 μm diamond plate. Ethanol and water‐free liquids were used during the entire sawing and grinding procedure. Once a thickness of about 30–50 μm was obtained, artificial cracks were introduced by running a high‐precision micro dicing saw (MicroAce Series 3, Dicing Saw) over the thin section. The width of the artificial cracks was the same as the sawing blade thickness, which was around 260 μm. Micrographs of the initial state of the thin sections were acquired by stereomicroscopy (Leica MZ6, Nussloch, Germany). To trigger the self‐healing mechanism of cement, thin sections were kept exposed to the atmosphere in a high‐humidity chamber (RH>95%) and they were examined after 28 days. The samples were not isolated from the surrounding environment in order to facilitate acceleration of the self‐healing mechanism triggered by the interaction with CO_2_. The analysis and self‐healing capacity assessment of the thin sections were based on image analysis of the micrographs. Micrographs of the entire crack were collected as soon as the crack was induced as well as after 28 days of age. Since the area of the un‐filled crack was the brightest portion of the micrographs, it could be segmented based on its grey‐scale value (GSV) and then quantified through ‘Analyze’ tools of the freeware ImageJ. The self‐healing capacity (SHC) of the active thin section was calculated as follows (1):

$$SHC\left[\%\right]=\left(A_{i}-A_{28d}\right)/A_{i}\;\times 100,$$

where *A_i_* is the area of the crack as soon as it was induced [mm^2^] and *A*_28d_ is the remaining area of the crack at 28 days of self‐healing incubation [mm^2^]. As a proof of concept, only a limited portion of the whole crack was analysed through this method in the present study.

## 3 RESULTS AND DISCUSSION

**FIGURE 2:**
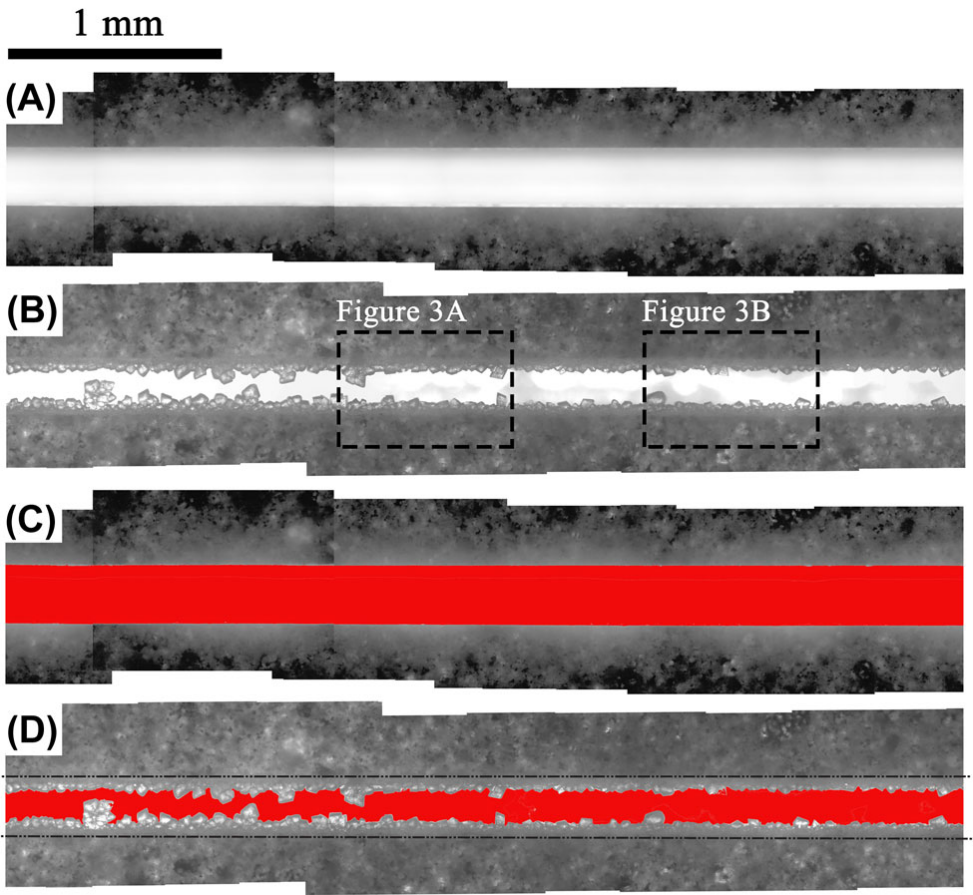
Microscope micrographs of active thin sections: (A) initial crack with no healing products; (B) crack at 28 days of self‐healing incubation (portions inside the dashed rectangles are reported in Figure 3A and B); (C) initial crack (segmented in red) for quantification of crack area, *A_i_*; (D) crack at 28 days (segmented in red) for quantification of self‐healing capacity, *A*_28d_. The walls of the initial crack area, *A_i_*, correspond to the black dotted lines

A typical self‐healing capacity of Portland cement paste can be visually observed by the active thin sections, as presented in Figure 2.

At 0 days of self‐healing incubation (i.e. 28 days after casting, Figure 2A), no self‐healing products were observed at the walls of the artificially‐induced crack. On the contrary, at 28 days of self‐healing incubation (Figure 2B) growing crystals partially filling up the crack were observable. Overall, self‐healing products were visible along the whole crack, with some portions that look almost completely filled by the crystals. In Figure 2C and D, the area of the unfilled crack has been firstly segmented (highlighted in red) and then its area measured to assess the SHC of the active thin section, as reported in Table 1.

**TABLE 1:** Results of image analysis to quantify self‐healing capacity of Portland cement paste

*A_i_* [mm^2^]	*A*_28d_ [mm^2^]	SHC [%]
1.267	0.562	55.6

According to previous studies,^3^, ^4^, ^5^ autogenous self‐healing of Portland cement specimens with no stimulating agent is generally limited to 100–150 μm. Hence, since the artificial cracks induced in this study were 260 μm, a SHC equal to 55.6% seems to be reasonably in line with the literature.

**FIGURE 3:**
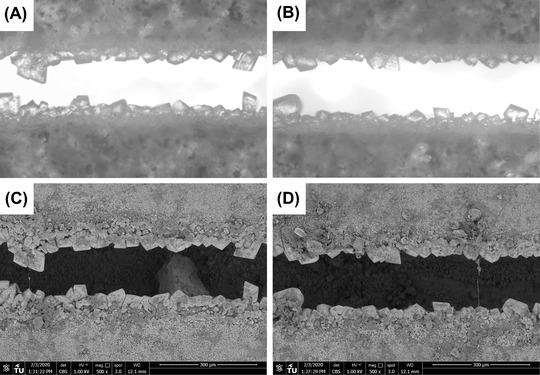
Microscope micrographs of partially healed portions of active thin sections: (A, B) crystals visible under plain polarised light; (C, D) crystals visible under ESEM/BSE mode

Environmental Scanning Electron Microscope (ESEM, FEI, Quanta FEG 650) micrographs of portions of the healed thin sections (dashed areas in Figure 2B) have been also collected and reported in Figure 3.

Looking at the rhombohedral morphology of the crystals forming at the sides of the crack and at the transparent colour when analysed through the light microscope, the healing products seem to be calcium carbonate crystals (e.g. calcite). The apparent good agreement between micrographs taken with the light microscope and the SEM allow to conduct image analysis for SHC quantification of the former ones, which detrimentally reduces the time of the experimental quantification. The degree at which the SHC results are sensitive to the micrographs that are considered will be quantitatively addressed in future studies.

## 4 CONCLUSIONS AND RECOMMENDATIONS

In this study, we proposed a methodology to evaluate and assess the self‐healing capacity of cementitious materials. As a proof of concept of this methodology, we evaluated the self‐healing mechanism of plain Portland cement paste triggered by carbonation in uncontrolled indoor conditions. Through microscopy and image analysis, a SHC equal to around 55% for a 260 μm wide crack after 28 days of self‐healing incubation was obtained. Through active thin sections, the autogenous self‐healing of cement paste due to carbonation can be observed and quantified during time non‐destructively. Theoretically, this methodology could be extended to observe self‐healing mechanisms of cementitious materials stimulated by embedded agents (i.e. vascular encapsulated polymers, crystals, bacteria, etc.).

When quantifying the SHC of engineered cementitious materials with currently available methodologies, it is always difficult to differentiate between the amount of healing products produced by any interaction with the surrounding environment and that triggered by the embedded healing agents. This differentiation would help to better evaluate the efficiency and actual SHC of the proposed systems. In the present proof of concept, no cover glass was placed at the top of the active thin section to facilitate the interaction between the matrix and the surrounding CO_2_. However, to observe the results of self‐healing due to added‐in agents only, isolation of the sample from the surrounding must be ensured. This might be achieved, among other methods, by placing a cover glass at the top of the section and isolating the glass sides, leaving a gap in the proximity of the crack mouth only. Also, keeping the section submerged in water would deny CO_2_ from reaching the sample surface. However, in this case the resulting SHC might be reduced since some healing agents (e.g. bacteria‐based ones) need the presence of O_2_ to be effective. Hence, more research on the applicability of active thin sections to evaluate stimulated self‐healing as well as comparing the efficiency of different engineered systems is in progress. However, based on the above‐mentioned characteristics of this method, applying active thin sections to evaluate the SHC of engineered self‐healing materials might bring valuable information, such as the 2D quantification of in‐time produced crystals as well as the locations at which these crystals precipitate.

It is worth mentioning again that the proposed methodology involves the preparation of thin sections subjected to cutting, grinding and polishing without prior embedding the sample in epoxy. Even though all the preparation steps have been conducted to minimise any potential contamination of the sample, particle pull‐outs, micro‐cracking and other artefacts are likely inevitable. For these and other reasons (e.g., the size of the sample, for instance), it might be that the self‐healing capacity measured through this method differs from the actual performance of the material exposed to different conditions or with different configurations. Nevertheless, this methodology could still be useful to observe some (still unclear) aspects related to the self‐healing mechanism of cementitious materials, such as the micro‐scale morphology of the growing crystals as well as the locations and conditions at which they get formed most likely. To this aim, further research is needed and is currently ongoing.
